# Nearest labelset using double distances for multi-label classification

**DOI:** 10.7717/peerj-cs.242

**Published:** 2019-12-09

**Authors:** Hyukjun Gweon, Matthias Schonlau, Stefan H. Steiner

**Affiliations:** 1 Department of Statistical and Actuarial Sciences, University of Western Ontario, London, Ontario, Canada; 2 Department of Statistics and Actuarial Science, University of Waterloo, Waterloo, Ontario, Canada

**Keywords:** Multi-label classification, Label correlations, Nearest neighbor

## Abstract

Multi-label classification is a type of supervised learning where an instance may belong to multiple labels simultaneously. Predicting each label independently has been criticized for not exploiting any correlation between labels. In this article we propose a novel approach, Nearest Labelset using Double Distances (*NLDD*), that predicts the labelset observed in the training data that minimizes a weighted sum of the distances in both the feature space and the label space to the new instance. The weights specify the relative tradeoff between the two distances. The weights are estimated from a binomial regression of the number of misclassified labels as a function of the two distances. Model parameters are estimated by maximum likelihood. *NLDD* only considers labelsets observed in the training data, thus implicitly taking into account label dependencies. Experiments on benchmark multi-label data sets show that the proposed method on average outperforms other well-known approaches in terms of 0/1 *loss*, and *multi-label accuracy* and ranks second on the *F-measure* (after a method called ECC) and on *Hamming loss* (after a method called *RF-PCT*).

## Introduction

In multi-label classification, an instance can belong to multiple labels at the same time. This is different from multi-class or binary classification, where an instance can only be associated with a single label. For example, a newspaper article talking about electronic books may be labelled with multiple topics such as business, arts and technology simultaneously. Multi-label classification has been applied in many areas of application including text (Schapire & Singer, 2000; Godbole & Sarawagi, 2004), image (Boutell et al., 2004; Zhang & Zhou, 2007), music (Li & Ogihara, 2003; Trohidis et al., 2008) and bioinformatics (Elisseeff & Weston, 2001). A labelset for an instance is the set of all labels that are associated with that instance.

Approaches for solving multi-label classification problems may be categorized into either problem transformation methods or algorithm adaptation methods (Tsoumakas & Katakis, 2007). Problem transformation methods transform a multi-label problem into one or more single-label problems. For the single-label classification problems, binary or multi-class classifiers are used. The results are combined and transformed back into a multi-label representation. Algorithm adaptation methods, on the other hand, modify specific learning algorithms directly for multi-label problems. Tsoumakas, Katakis & Vlahavas (2010), Madjarov et al. (2012) and Zhang & Zhou (2014) give overviews of multi-label algorithms and evaluation metrics.

In this article, we propose a new problem transformation approach to multi-label classification. Our proposed approach applies the nearest neighbor method to predict the label with the shortest distance in the feature space. However, because we have multiple labels, we additionally consider the shortest (Euclidean) distance in the label space where the input of the test instance in the label space consists of probability outputs obtained by independent binary classifiers. We then find the labelset that minimizes the expected label misclassification rate as a function of both feature space and label space distances, thus exploiting high-order interdependencies between labels. The nonlinear function is estimated using maximum likelihood.

The effectiveness of the proposed approach is evaluated with various multi-label data sets. Our experiments show that the proposed method performs on average better on standard evaluation metrics (*Hammmingloss*, 0∕1*loss*, *multi*-*label accuracy* and the *F*-*measure*) than other commonly used algorithms.

The rest of this article is organized as follows: in ‘Related work’ we review previous work on multi-label classification. In ‘The nearest labelset using double distances approach’, we present the details of the proposed method. In ‘Experimental Evaluation’, we report on experiments that compare the proposed method with other algorithms on standard metrics. In ‘Discussion’ we discuss the results. In ‘Conclusion’, we draw conclusions.

## Related Work

In this section, we briefly review the multi-label approaches that are existing competitors to the proposed method.

There are several approaches to classifying multi-label data. The most common approach, binary relevance (*BR*) (Zhang & Zhou, 2005; Tsoumakas & Katakis, 2007), transforms a multi-label problem into separate binary problems. That is, using training data, *BR* constructs a binary classifier for each label independently. For a test instance, the prediction set of labels is obtained simply by combining the individual binary results. In other words, the predicted labelset is the union of the results predicted from the *L* binary models. This approach requires one binary model for each label. The method has been adapted in many domains including text (Gonçalves & Quaresma, 2003), music (Li & Ogihara, 2003) and images (Boutell et al., 2004). One drawback of the basic binary approach is that it does not account for any correlation that may exist between labels, because the labels are modelled independently. Taking correlations into account is often critical for prediction in multi-label problems (Godbole & Sarawagi, 2004; Ji et al., 2008).

Subset-Mapping (*SMBR*) (Schapire & Singer, 1999; Read et al., 2011) is a method related to *BR*. For a new instance, first labels are predicted by the binary outputs of *BR*. Then, final prediction is made by the training labelset with the shortest *Hamming* distance to the predicted labelset. *SMBR* makes predictions by selecting labelsets observed in the training data. *SBMR* is a nearest neighbor approach in the label space—from the set of predicted labels to the sets of labels observed in the training data—with *Hamming* distance as the distance metric.

An extension of binary relevance is Classifier Chain (*CC*) (Read et al., 2011). *CC* fits labels sequentially using binary classifiers. Labels already predicted are included as features in subsequent classifiers until all labels have been fit. Including previous predictions as features “chains” the classifiers together and also takes into account potential label correlations. However, the order of the labels in a chain affects the predictive performances. Read et al. (2011) also introduced the ensemble of classifier chains (*ECC*), where multiple *CC* are built with re-sampled training sets. The order of the labels in each *CC* is randomly chosen. The prediction label of an *ECC* is obtained by the majority vote of the *CC* models.

Label Powerset learning (*LP*) transforms a multi-label classification into a multi-class problem (Tsoumakas & Katakis, 2007). In other words, *LP* treats each labelset as a single label. The transformed problem requires a single classifier. Although *LP* captures correlations between labels, the number of classes in the transformed problem increases exponentially with the number of original labels. *LP* learning can only choose observed labelsets for predictions (Tsoumakas & Katakis, 2007; Read, Pfahringer & Holmes, 2008).

The random k-labelsets method, (*RAKEL*) (Tsoumakas & Vlahavas, 2007), is a variation on the *LP* approach. In a multi-label problem with *L* different labels, *RAKEL* employs *m* multi-class models each of which considers *k*(≤*L*) randomly chosen labels, rather than the entire labelset. For a test instance, the prediction labelset is obtained by the majority vote of the results based on the *m* models. *RAKEL* overcomes the problem that the number of multinomial classes increases exponentially as a function of the number of labels. It also considers interdependencies between labels by using multi-class models with subsets of the labels.

A hierarchy of multi-label classifiers (*HOMER*) (Tsoumakas, Katakis & Vlahavas, 2008) constructs a tree-shaped hierarchy by partitioning the labels recursively into smaller disjoint subsets (i.e., nodes) using a balanced clustering algorithm, which extends the *k* means algorithm with an additional constraint on the size of each cluster. After that, *HOMER* constructs a multi-label classifier for the labelsets in each node. For the prediction of a new instance, *HOMER* follows a top-down recursive process from the root. A classifier on a non-root node is called only if the prediction of its parent node is positive. The final labelset is determined by the positive leaves (i.e., labels) whose parent nodes are all positive.

A popular lazy learning algorithm based on the *k* Nearest Neighbours (*kNN*) approach is *MLKNN* (Zhang & Zhou, 2007). Like other *kNN*-based methods, *MLKNN* identifies the *k* nearest training instances in the feature space for a test instance. Then for each label, *MLKNN* estimates the prior and likelihood for the number of neighbours associated with the label. Using Bayes theorem, *MLKNN* calculates the posterior probability from which a prediction is made.

**Figure 1:**
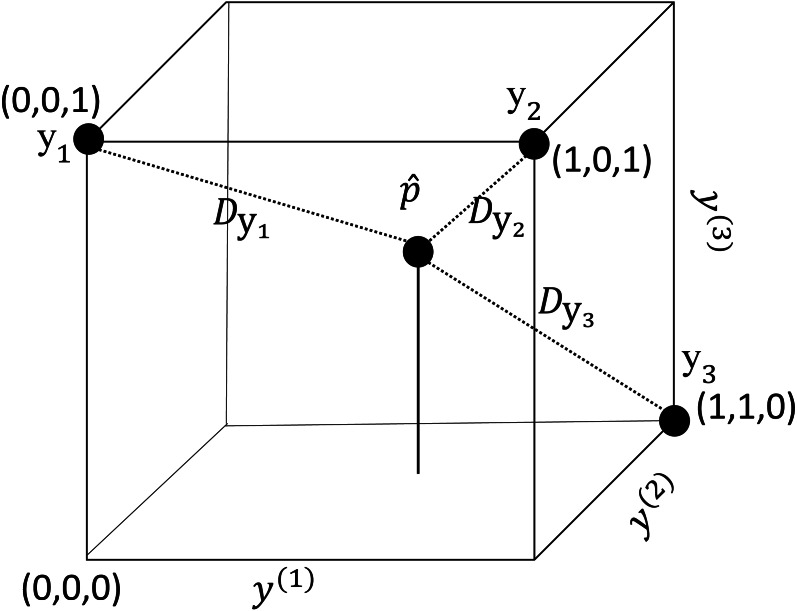
An illustration of the label space when *L* = 3. Each vertex represents a labelset. The inner point represents a fitted vector of an instance. ${D}_{{\vec{y}}_{i}}$ represents the distance between $\hat {\vec{p}}$ and ${\vec{y}}_{i}$.

The Conditional Bernoulli Mixtures (*CBM*) (Li et al., 2016) approach transforms a multi-label problem into a mixture of binary and multi-class problems. *CBM* divides the feature space into *K* regions and learns a multi-class classifier for the regional components as well as binary classifiers in each region. The posterior probability for a labelset is obtained by mixing the multi-class and multiple binary classifiers. The model parameters are estimated using the Expectation Maximization algorithm.

Multi-target classification approaches may also be used for multi-label classification. A number of multi-target learning methods use the predictive clustering tree (*PCT*) (Blockeel, Raedt & Ramon, 1998) as the base classifier. Random forest of predictive clustering trees (*RF*- *PCT*) (Kocev et al., 2007) has been shown to be competitive (Madjarov et al., 2012). *RF*-*PCT* is a tree-based ensemble method using *PCT*s as base classifiers. Different *PCT*s are constructed from different bootstrap samples and random subsets of the features.

## The Nearest Labelset Using Double Distances Approach

### Hypercube view of a multi-label problem

In multi-label classification, we are given a set of possible output labels $\mathcal{L}=\{1,2,&hellip; ,L\}$. Each instance with a feature vector $\vec{x}\in {\mathbb{R}}^{d}$ is associated with a subset of these labels. Equivalently, the subset can be described as $\vec{y}=({y}^{(1)},{y}^{(2)},\ldots ,{y}^{(L)})$, where *y*^(*i*)^ = 1 if label *i* is associated with the instance and *y*^(*i*)^ = 0 otherwise. A multi-label training data set is described as $T=\{({\vec{x}}_{i},{\vec{y}}_{i})\},$i=1 , {2, …, *N*}.

Any labelset $\vec{y}$ can be described as a vertex in the *L*-dimensional unit hypercube (Tai & Lin, 2012). Each component *y*^(*i*)^ of $\vec{y}$ represents an axis of the hypercube. As an example, Fig. 1 illustrates the label space of a multi-label problem with three labels (*y*^(1)^, *y*^(2)^, *y*^(3)^).

Assume that the presence or absence of each label is modeled independently with a probabilistic classifier. For a new instance, the classifiers provide the probabilities, *p*^(1)^, …, *p*^(*L*)^, that the corresponding labels are associated with the instance. Using the probability outputs, we may obtain a *L*-dimensional vector $\hat {\vec{p}}=({p}^{(1)},{p}^{(2)},\ldots ,{p}^{(L)})$. Every element of $\hat {\vec{p}}$ has a value from 0 to 1 and the vector $\hat {\vec{p}}$ is an inner point in the hypercube (see Fig. 1). Given $\hat {\vec{p}}$ the prediction task is completed by assigning the inner point to a vertex of the cube.

For the new instance, we may calculate the Euclidean distance, ${D}_{{\vec{y}}_{i}}$, between $\hat {\vec{p}}$ and each ${\vec{y}}_{i}$ (i.e., the labelset of the *i*th training instance). In Fig. 1, three training instances ${\vec{y}}_{1}$, ${\vec{y}}_{2}$ and ${\vec{y}}_{3}$ and the corresponding distances are shown. A small distance ${D}_{{\vec{y}}_{i}}$ indicates that ${\vec{y}}_{i}$ is likely to be the labelset for the new instance.

### Nearest labelset using double distances (*NLDD*)

In addition to computing the distance in the label space, ${D}_{{\vec{y}}_{i}}$, we may also obtain the (Euclidean) distance in the feature space, denoted by ${D}_{{\vec{x}}_{i}}$. The proposed method, *NLDD*, uses both ${D}_{\vec{x}}$ and ${D}_{\vec{y}}$ as predictors to find a training labelset that minimizes the expected loss. For each test instance, we define loss as the number of misclassified labels out of *L* labels. The expected loss is then *Lθ* where $\theta =g({D}_{\vec{x}},{D}_{\vec{y}})$ represents the probability of misclassifying each label. The predicted labelset, ${\hat {\vec{y}}}^{\ast }$, is the labelset observed in the training data that minimizes the expected loss:

(1) \begin{eqnarray*}{\hat {\vec{y}}}^{\ast }=\argmin _{\vec{y}\in T} Lg({D}_{\vec{x}},{D}_{\vec{y}})\end{eqnarray*}

The loss follows a binomial distribution with *L* trials and a parameter *θ*. We model $\theta =g({D}_{\vec{x}},{D}_{\vec{y}})$ using binomial regression. Specifically,

(2) \begin{eqnarray*}\log \nolimits \left( \frac{\theta }{1-\theta } \right) ={\beta }_{0}+{\beta }_{1}{D}_{\vec{x}}+{\beta }_{2}{D}_{\vec{y}}\end{eqnarray*}

where *β*_0_, *β*_1_ and *β*_2_ are the model parameters. Greater values for *β*_1_ and *β*_2_ imply that *θ* becomes more sensitive to the distances in the feature and label spaces, respectively. The misclassification probability decreases as ${D}_{\vec{x}}$ and ${D}_{\vec{y}}$ approach zero.

A test instance with ${D}_{\vec{x}}={D}_{\vec{y}}=0$ has a duplicate instance in the training data (i.e., with identical features). The predicted probabilities for the test instance are either 0 or 1 and the match the labels of the duplicate training observation. For such a “double”-duplicate instance (i.e., ${D}_{\vec{x}}={D}_{\vec{y}}=0$), the probability of misclassification is 1∕(1 + *e*^−*β*_0_^) > 0. As expected, the uncertainty of a test observation with a “double-duplicate” training observation is greater than zero. This is not surprising: duplicate training observations do not necessarily have the same response, and neither do double-duplicate observations.

The model in Eq. (2) implies $g({D}_{\vec{x}},{D}_{\vec{y}})=1/(1+{e}^{-({\beta }_{0}+{\beta }_{1}{D}_{\vec{x}}+{\beta }_{2}{D}_{\vec{y}})})$. Because $\log \left( \frac{\theta }{1-\theta } \right) $ is a monotone transformation of *θ* and *L* is a constant, the minimization problem in (1) is equivalent to

(3) \begin{eqnarray*}{\hat {\vec{y}}}^{\ast }=\argmin _{(\vec{x},\vec{y})\in T} {\beta }_{1}{D}_{\vec{x}}+{\beta }_{2}{D}_{\vec{y}}\end{eqnarray*}

That is, *NLDD* predicts by choosing the labelset of the training instance that minimizes the weighted sum of the distances. For prediction, the only remaining issue is how to estimate the weights.

 
________________________________________________________________________________________________________________________________________________________________ 
Algorithm 1 The training process of NLDD 
________________________________________________________________________________________________________________________________________________________________ 
  Input: training data T, number of labels L 
  Output: probabilistic classifiers h(i), binomial regression g 
  Split T into T1 and T2 
  for i = 1 to L do 
      train probabilistic classifier h(i) based on T 
      train probabilistic classifier h(i)∗ based on T1 
  end for 
  S,W ←∅ 
  for each instance in T2 do 
      obtain ˆ p = (h(1)∗ (x),...,h(L)∗ (x)) 
      for each instance in T1 do 
          compute Dx and Dy 
          W ← W ∪ (Dx,Dy) 
      end for 
      find m1,m2 ∈ W 
      update S ← S ∪{m1,m2} 
  end for 
  Fit log (θ __1−θ) = β0 + β1Dx + β2Dy to S 
  Obtain g : S → ˆ θ =   e ˆf1+e ˆf where  ˆ f= ˆ β0 + ˆ β1Dx + ˆ β2Dy 
________________________________________________________________________________________________________________________________________________________________    

 
_______________________________________________________________________________________________________ 
Algorithm 2 The classification process of NLDD 
_______________________________________________________________________________________________________ 
Input: new instance x, binomial model g, probabilistic classifiers h(i), training data T of 
size N 
Output: multi-label classification vector ˆ y 
for j = 1 to N do 
    compute ˆ p = (h(1)(x),...,h(L)(x)) 
    compute Dxj  and Dyj 
    obtain ˆ θj ← g(Dxj,Dyj) 
end for 
return ˆ y ← argmin  yj∈T   ˆ θj 
________________________________________________________________________________________________________ 

### Estimating the relative weights of the two distances

The weights *β*_0_, *β*_1_ and *β*_2_ can be estimated using binomial regression. Binomial regression can be fit by running separate logistic regressions, one for each of the *L* labels. To run the regressions ${D}_{\vec{x}}$ and ${D}_{\vec{y}}$ need to be computed on the training data. For this purpose we split the training data (*T*) equally into an internal training data set, *T*_1_, and an internal validation data set, *T*_2_.^1^ 1The dataset is split equally for training and testing. An unequal split is not desirable: adding more instances to the internal training set may improve the performance of individual probabilistic classifiers. However, this would lead to the decrease of the number of distance pairs that are needed for the binomial regression modeling (# distance pairs = 2(# instance in the internal validation set)). That is, reducing the size of the validation set will decrease the amount of data used for binomial regression.We next fit a binary classifier to each of the *L* labels separately and obtain the labelset predictions (i.e., probability outcomes) for the instances in *T*_2_. In principle, each observation in *T*_2_ can be paired with each observation in *T*_1_, creating a $({D}_{\vec{x}},{D}_{\vec{y}})$ pair, and the regressions can be run on all possible pairs. Note that matching any single instance in *T*_2_ to those in *T*_1_ results in *N*∕2 distance pairs. However, most of the pairs are uninformative because the distance in either the feature space or the label space is very large. Since candidate labelsets for the final prediction will have a small ${D}_{\vec{y}}$ and a small ${D}_{\vec{x}}$, it is reasonable to focus more on the behavior of the loss especially at small values of ${D}_{\vec{x}}$ and ${D}_{\vec{y}}$ than considering the loss at the entire range of the distances. Moreover, since *T*_2_ contains *N*∕2 instances, the number of possible pairs is potentially large (*N*^2^/4). Therefore, to reduce computational complexity, for each instance we only identify two pairs: the pair with the smallest distance in $\vec{x}$ and the pair with the smallest distance in $\vec{y}$. In case of ties in one distance, the pair with the smallest value in the other distance is chosen. More formally, we identify the first pair *m*_*i*_1__ by

\begin{eqnarray*}{m}_{{i}_{1}}=\argmin _{({D}_{x},{D}_{y})\in {W}_{ix}}{D}_{y} \end{eqnarray*}

where *W*_*ix*_ is the set of pairs that are tied; i.e., that each corresponds to the minimum distance in *D*_*x*_. Similarly, the second pair *m*_*i*_2__ is found by

**Figure 2:**
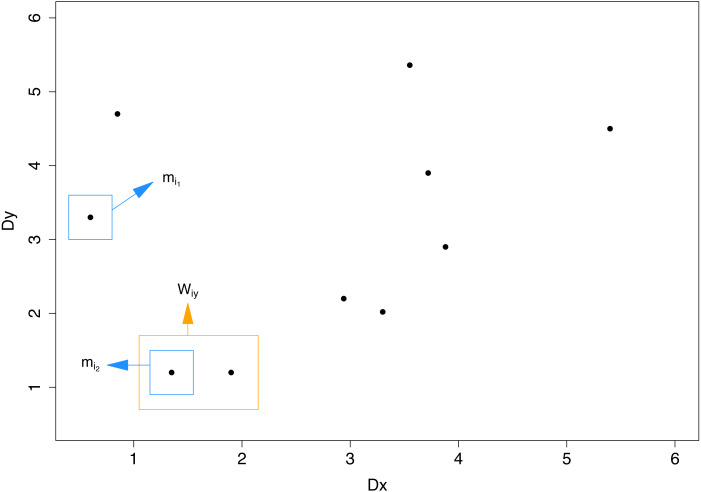
An illustration of how to identify *m*_*i*_1__ and *m*_*i*_2__ for *N* = 20.*T*_1_ and *T*_2_ contain 10 instances each. The 10 points in the scatter plot were obtained by calculating *D*_*x*_ and *D*_*y*_ between a single instance in *T*_2_ and the 10 instances in *T*_1_. In this example two points have the lowest distance in *D*_*y*_ and are candidates for *m*_*i*_2__. Among the candidates, the point with the lowest *D*_*x*_ is chosen.

\begin{eqnarray*}{m}_{{i}_{2}}=\argmin _{({D}_{x},{D}_{y})\in {W}_{iy}}{D}_{x}. \end{eqnarray*}

where *W*_*iy*_ is the set of labels that are tied with the minimal distance in *D*_*y*_. Figure 2 illustrates an example of how to identify *m*_*i*_1__ and *m*_*i*_2__ for *N* = 20. Our goal was to identify the instance with the smallest distance in $\vec{x}$ and the instance with the smallest distance in $\vec{y}$. Note that *m*_*i*_1__ and *m*_*i*_2__ may be the same instance. If we find a single instance that minimizes both distances, we use just that instance. (A possible duplication of that instance is unlikely to make any difference in practice).

The two pairs corresponding to the *i*th instance in *T*_2_ are denoted as the set ${S}_{i}= \left\{ {m}_{{i}_{1}},{m}_{{i}_{2}} \right\} $, and their union for all instances is denoted as $S={\mathop{\bigcup }\nolimits }_{i=1}^{N/2}{S}_{i}$. The binomial regression specified in Eq. (2) is performed on the $2 \frac{N}{2} =N$ instances in *S*. Algorithm 1 outlines the training procedure.

For the classification of a new instance, we first obtain $\hat {\vec{p}}$ using the probabilistic classifiers fitted to the training data *T*. ${D}_{{\vec{x}}_{j}}$ and ${D}_{{\vec{y}}_{j}}$ are obtained by matching the instance with the *j*th training instance. Using the *MLEs* ${\hat {\beta }}_{0}$, ${\hat {\beta }}_{1}$ and ${\hat {\beta }}_{2}$, we calculate ${\hat {\theta }}_{j}= \frac{{e}^{{\hat {f}}_{j}}}{1+{e}^{{\hat {f}}_{j}}} $ where ${\hat {f}}_{j}={\hat {\beta }}_{0}+{\hat {\beta }}_{1}{D}_{{\vec{x}}_{j}}+{\hat {\beta }}_{2}{D}_{{\vec{y}}_{j}}$. The final prediction of the new instance is obtained by

\begin{eqnarray*}\hat {\vec{y}}=\argmin _{{\vec{y}}_{j}\in T} \hat {E}(loss)=\argmin _{{\vec{y}}_{j}\in T} {\hat {\theta }}_{j}. \end{eqnarray*}

The second equality holds because $\hat {E}(loss)=L\hat {\theta }$ and *L* is a constant. As in *LP*, *NLDD* chooses a training labelset as the predicted vector. Algorithm 2 outlines the classification procedure.

The training time of *NLDD* is *O*(*L*(*f*(*d*, *N*) + *f*(*d*, *N*∕2) + *g*(*d*, *N*∕2)) + *N*^2^(*d* + *L*) + *Nlog*(*k*)) where *O*(*f*(*d*, *N*)) is the complexity of each binary classifier with *d* features and *N* training instances, *O*(*g*(*d*, *N*∕2)) is the complexity for predicting each label for *T*_2_, *N*^2^(*d* + *L*) is the complexity for obtaining the distance pairs for the regression and *O*(*Nlog*(*k*)) is the complexity for fitting a binomial regression. *T*_1_ and *T*_2_ have *N*∕2 instances respectively. *O*(*Lf*(*d*, *N*∕2)) is the complexity for fitting binary classifiers using *T*_1_ and obtaining the probability results for *T*_2_ takes *O*(*Lg*(*d*, *N*∕2)). For each instance of *T*_2_, we obtain *N*∕2 numbers of distance pairs. This has complexity *O*((*N*∕2)(*d* + *L*)). Since there are *N*∕2 instances, overall it takes *O*((*N*∕2)(*N*∕2)(*d* + *L*)) or *O*(*N*^2^(*d* + *L*)) when omitting the constant. Among the *N*∕2 pairs for each instance of *T*_2_, we only identify at most 2 pairs. This implies *N*∕2 ≤ *s* ≤ *N* where *s* is the number of elements in *S*. Each iteration of the Newton–Raphson method has a complexity of *O*(*N*). For *k*-digit precision complexity *O*(*logk*) is required (Ypma, 1995). Combined, the complexity for estimating the parameters with *k*-digit precision is *O*(*Nlog*(*k*)). In practice, however, this term is dominated by *N*^2^(*d* + *L*) as we can set *k* <  < *N*.

## Experimental Evaluation

In this section we compare different multi-label algorithms on nine data sets. We next introduce the data sets and the evaluation measures and then present the results of our experiments.

### Data sets

We evaluated the proposed approach using nine commonly used multi-label data sets from different domains. Table 1 shows basic statistics for each data set including its domain, numbers of labels and features. In the text data sets, all features are categorical (i.e., binary). The last column “lcard”, short for label cardinality, represents the average number of labels associated with an instance. The data sets are ordered by (|*L*|⋅|*X*|⋅|*E*|).

The *emotions* data set (Trohidis et al., 2008) consists of pieces of music with rhythmic and timbre features. Each instance is associated with up to 6 emotion labels such as “sad-lonely”, “amazed-surprised” and “happy-pleased”. The *scene* data set (Boutell et al., 2004) consists of images with 294 visual features. Each image is associated with up to 6 labels including “mountain”, “urban” and “beach”. The *yeast* data set (Elisseeff & Weston, 2001) contains 2,417 yeast genes in the Yeast Saccharomyces Cerevisiae. Each gene is represented by 103 features and is associated with a subset of 14 functional labels. The *medical* data set consists of documents that describe patient symptom histories. The data were made available in the Medical Natural language Processing Challenge in 2007. Each document is associated with a set of 45 disease codes. The *slashdot* data set consists of 3,782 text instances with 22 labels obtained from Slashdot.org. The *enron* data set (Klimt & Yang, 2004) contains 1,702 email messages from the Enron corporation employees. The emails were categorized into 53 labels. The *ohsumed* data set (Hersh et al., 1994) is a collection of medical research articles from MEDLINE database. We used the same data set as in (Read et al., 2011) that contains 13,929 instances and 23 labels. The *tmc*2007 data set (Srivastava & Zane-Ulman, 2005) contains 28,596 aviation safety reports associated with up to 22 labels. Following Tsoumakas, Katakis & Vlahavas (2011), we used a reduced version of the data set with 500 features. The *bibtex* data set (Katakis, Tsoumakas & Vlahavas, 2008) consists of 7,395 bibtex entries for automated tag suggestion. The entries were classified into 159 labels. All data sets are available online at: MULAN (http://mulan.sourceforge.net/datasets-mlc.html) and MEKA (http://meka.sourceforge.net/#datasets).

**Table 1:** Multi-label data sets and their associated characteristics. Label cardinality (lcards) is the average number of labels associated with an instance.

Name	Domain	Labels (|*L*|)	Features (|*X*|)	Examples (|*E*|)	Lcards
emotions	music	6	72	593	1.87
scene	image	6	294	2,407	1.07
yeast	biology	14	103	2,417	4.24
medical	text	45	1,449	978	1.25
slashdot	text	22	1,079	3,782	1.18
enron	text	53	1,001	1,702	3.37
ohsumed	text	23	1,002	1,3929	1.66
tmc2007	text	22	500	2,8596	2.16
bibtex	text	159	1,836	7,395	2.40

### Evaluation metrics

Multi-label classifiers can be evaluated with various loss functions. Here, four of the most popular criteria are used: *Hammingloss*, 0∕1*loss*, *multi*-*label accuracy* and *F*-*measure*. These criteria are defined in the following paragraphs.

Let *L* be the number of labels in a multi-label problem. For a particular test instance, let $\vec{y}=({y}^{(1)},\ldots ,{y}^{(L)})$ be the labelset where *y*^(*j*)^ = 1 if the *j*th label is associated with the instance and 0 otherwise. Let $\hat {\vec{y}}=({\hat {y}}^{(1)},\ldots ,{\hat {y}}^{(L)})$ be the predicted values obtained by any machine learning method. *Hammingloss* refers to the percentage of incorrect labels. The *Hammingloss* for the instance is

\begin{eqnarray*}\text{}Hamming loss\text{}=1- \frac{1}{L} \sum _{j=1}^{L}\mathbb{1}\{{y}^{(j)}={\hat {y}}^{(j)}\} \end{eqnarray*}

where *1* is the indicator function. Despite its simplicity, the *Hammingloss* may be less discriminative than other metrics. In practice, an instance is usually associated with a small subset of labels. As the elements of the *L*-dimensional label vector are mostly zero, even the empty set (i.e., zero vector) prediction may lead to a decent *Hammingloss*.

The 0∕1*loss* is 0 if all predicted labels match the true labels and 1 otherwise. Hence,

\begin{eqnarray*}\text{}0/1 loss\text{}=1-\mathbb{1}\{\vec{y}=\hat {\vec{y}}\}. \end{eqnarray*}

Compared to other evaluation metrics, 0∕1 *loss* is strict as all the *L* labels must match to the true ones simultaneously. The *multi*-*label accuracy* (Godbole & Sarawagi, 2004) (also known as the *Jaccard* index) is defined as the number of labels counted in the intersection of the predicted and true labelsets divided by the number of labels counted in the union of the labelsets. That is,

\begin{eqnarray*}Multi\text{-}labelaccuracy= \frac{{|}\vec{y}\cap \hat {\vec{y}}{|}}{{|}\vec{y}\cup \hat {\vec{y}}{|}} . \end{eqnarray*}

The *multi*-*label accuracy* measures the similarity between the true and predicted labelsets.

The *F*-*measure* is the harmonic mean of precision and recall. The *F*-*measure* is defined as

\begin{eqnarray*}F\text{-}measure= \frac{2{|}\vec{y}\cap \hat {\vec{y}}{|}}{{|}\vec{y}{|}+{|}\hat {\vec{y}}{|}} . \end{eqnarray*}

The metrics above were defined for a single instance. On each metric, the overall value for an entire test data set is obtained by averaging out the individual values.

### Experimental setup

We compared our proposed method against *BR*, *SMBR*, *ECC*, *RAKEL*, *HOMER*, *RF*-*PCT*, *MLKNN* and *CBM*. To train multi-label classifiers, the parameters recommended by the authors were used, since they appear to give the best (or comparable to the best) performance in general. In the case of *MLKNN*, we set the number of neighbors and the smoothing parameter to 10 and 1 respectively. For *RAKEL*, we set the number of separate models to 2*L* and the size of each sub-labelset to 3. For *ECC*, the number of *CC* models for each ensemble was set to 10. For *HOMER*, the number of clusters was set to 3 as used in Liu et al. (2015). On the larger data sets (*ohsumed*, *tmc*2007 and *bibtex*), we fit *ECC* using reduced training data sets (75% of the instances and 50% of the features) as suggested in Read et al. (2011). On the same data sets, we ran *NLDD* using 70% of the training data to reduce redundancy in learning.

For *NLDD*, we used support vector machines (*SVM*) (Vapnik, 2000) as the base classifier on unscaled variables with a linear kernel and tuning parameter *C* = 1. The *SVM* scores were converted into probabilities using Platt’s method (Platt, 2000). The analysis was conducted in *R* (R Core Team, 2014) using the *e*1071 package (Meyer et al., 2014) for *SVM*. For the data sets with less than 5,000 instances 10-fold cross validations (*CV*) were performed. On the larger data sets, we used 75/25 train/test splits. For fitting binomial regression models, we divided the training data sets at random into two parts of equal sizes.

For *RF*-*PCT*, we used the *Clus* (http://clus.sourceforge.net) system. In the pre-pruning strategy of *PCT*, the significance level for the F-test was automatically chosen from {0.001, 0.005, 0.01, 0.05, 0.1, 0.125} using a reserved prune-set.

For *CBM*, we used the authors’ Java program (https://github.com/cheng-li/pyramid). The default settings (e.g., logistic regression and 10 iterations for the *EM* algorithm) were used on non-large data sets. For the large data sets *tmc*2007 and *bibtex*, the number of iterations was set to 5 and random feature reduction was applied as suggested by the developers. On each data set we used the train/test split recommended at their website (https://github.com/cheng-li/pyramid).

To test the hypothesis that all classifiers perform equally, we used the Friedman test as recommended by Demšar (2006). We then compared *NLDD* with each of the other methods using Wilcoxon signed-rank tests (Wilcoxon, 1945). We adjusted *p*-values for multiple testing using Hochberg’s method (Hochberg, 1988).

In *NLDD*, when calculating distances in the feature spaces we used the standardized features so that no particular features dominated distances. For a numerical feature variable *x*, the standardized variable *z* is obtained by $z=(x-\bar {x})/\text{sd}(x)$ where $\bar {x}$ and sd(*x*) are the mean and standard deviation of *x* in the training data.

### Results

Tables 2 to 5 summarize the results in terms of *Hammingloss*, 0∕1*loss*, *multi*-*label accuracy* and *F*-*measure*, respectively. We also ranked the algorithms for each metric.

**Table 2:** *Hammingloss* (lower is better) averaged over 10 cross validations (with ranks in parentheses). The data sets are ordered as in Table 1.

Data	*BR*	*SMBR*	*NLDD*	*ECC*	*RAKEL*	*HOMER*	*RF*-*PCT*	*MLKNN*	*CBM*
emotions	0.196 (4)	0.200 (5)	0.190 (2)	0.201 (6)	0.195 (3)	0.211 (7)	0.188 (1)	0.265 (8)	0.337 (9)
scene	0.104 (7)	0.130 (9)	0.095 (5)	0.094 (4)	0.089 (2)	0.109 (8)	0.088 (1)	0.090 (3)	0.095 (6)
yeast	0.199 (5)	0.205 (6)	0.190 (1)	0.206 (7)	0.196 (4)	0.254 (9)	0.192 (2)	0.195 (3)	0.213 (8)
medical	0.010 (3)	0.011 (6)	0.010 (4)	0.009 (2)	0.010 (5)	0.014 (8)	0.012 (7)	0.015 (9)	0.009 (1)
slashdot	0.047 (5)	0.054 (8)	0.045 (4)	0.047 (6)	0.044 (2)	0.055 (9)	0.044 (3)	0.052 (7)	0.044 (1)
enron	0.058 (9)	0.056 (8)	0.055 (5)	0.052 (3)	0.055 (6)	0.055 (7)	0.046 (1)	0.053 (2)	0.053 (4)
ohsumed	0.067 (5)	0.072 (7)	0.061 (3)	0.074 (8)	0.060 (2)	0.079 (9)	0.057 (1)	0.070 (6)	0.064 (4)
tmc2007	0.058 (3)	0.059 (4)	0.058 (2)	0.063 (6)	0.059 (5)	0.065 (7)	0.053 (1)	0.071 (9)	0.070 (8)
bibtex	0.016 (8)	0.015 (7)	0.013 (1)	0.015 (5)	0.015 (6)	0.021 (9)	0.014 (2)	0.014 (4)	0.014 (3)
av. ranks	5.4	6.7	3.0	5.2	3.8	8.1	2.1	5.7	4.9

**Table 3:** 0∕1*loss* (lower is better) averaged over 10 cross validations (with ranks in parentheses). The loss is 0 if a predicted labelset matches the true labelset exactly and 1 otherwise.

Data	*BR*	*SMBR*	*NLDD*	*ECC*	*RAKEL*	*HOMER*	*RF*- *PCT*	*MLKNN*	*CBM*
emotions	0.718 (7)	0.708 (5)	0.690 (3)	0.710 (6)	0.679 (2)	0.695 (4)	0.662 (1)	0.885 (9)	0.798 (8)
scene	0.467 (9)	0.424 (7)	0.319 (1)	0.351 (3)	0.364 (4)	0.377 (6)	0.436 (8)	0.370 (5)	0.321 (2)
yeast	0.894 (8)	0.818 (6)	0.748 (1)	0.798 (3)	0.813 (4)	0.977 (9)	0.821 (7)	0.818 (5)	0.751 (2)
medical	0.319 (6)	0.307 (4)	0.279 (2)	0.302 (3)	0.319 (5)	0.321 (7)	0.392 (8)	0.494 (7)	0.226 (1)
slashdot	0.645 (7)	0.625 (5)	0.523 (2)	0.600 (4)	0.628 (6)	0.597 (3)	0.797 (8)	0.939 (9)	0.513 (1)
enron	0.907 (8)	0.877 (4)	0.866 (2)	0.879 (5)	0.900 (6)	0.906 (7)	0.871 (3)	0.959 (9)	0.830 (1)
ohsumed	0.799 (7)	0.787 (6)	0.720 (1)	0.820 (8)	0.774 (4)	0.776 (5)	0.768 (3)	0.949 (9)	0.734 (2)
tmc2007	0.706 (5)	0.704 (4)	0.702 (2)	0.732 (7)	0.703 (3)	0.730 (6)	0.645 (1)	0.773 (9)	0.736 (8)
bibtex	0.850 (6)	0.820 (3)	0.805 (2)	0.839 (4)	0.841 (5)	0.899 (7)	0.913 (8)	0.944 (9)	0.782 (1)
av. ranks	6.9	4.9	1.8	4.8	4.3	6.0	5.2	8.1	2.9

**Table 4:** *Multi*- *labelaccuracy* (higher is better) averaged over 10 cross validations (with ranks in parentheses).

Data	*BR*	*SMBR*	*NLDD*	*ECC*	*RAKEL*	*HOMER*	*RF*- *PCT*	*MLKNN*	*CBM*
emotions	0.525 (7)	0.547 (6)	0.562 (2)	0.559 (3)	0.555 (4)	0.579 (1)	0.552 (5)	0.325 (9)	0.403 (8)
scene	0.636 (8)	0.651 (7)	0.742 (1)	0.699 (4)	0.699 (3)	0.692 (5)	0.587 (9)	0.690 (6)	0.718 (2)
yeast	0.499 (8)	0.509 (7)	0.546 (1)	0.543 (2)	0.519 (4)	0.431 (9)	0.515 (5)	0.510 (6)	0.522 (3)
medical	0.766 (6)	0.768 (5)	0.799 (2)	0.793 (3)	0.764 (7)	0.769 (4)	0.675 (8)	0.579 (9)	0.817 (1)
slashdot	0.452 (7)	0.469 (5)	0.535 (2)	0.507 (3)	0.458 (6)	0.495 (4)	0.216 (8)	0.069 (9)	0.550 (1)
enron	0.397 (8)	0.423 (5)	0.412 (6)	0.471 (1)	0.409 (7)	0.427 (4)	0.453 (2)	0.318 (9)	0.430 (3)
ohsumed	0.385 (7)	0.397 (5)	0.435 (2)	0.432 (3)	0.394 (6)	0.422 (4)	0.341 (8)	0.080 (9)	0.492 (1)
tmc2007	0.575 (3)	0.578 (2)	0.570 (6)	0.567 (7)	0.571 (5)	0.574 (4)	0.607 (1)	0.472 (9)	0.519 (8)
bibtex	0.326 (6)	0.339 (3)	0.351 (2)	0.332 (5)	0.334 (4)	0.256 (7)	0.159 (8)	0.128 (9)	0.376 (1)
av. ranks	6.7	5.0	2.7	3.4	5.1	4.7	6.0	8.3	3.1

**Table 5:** *F*-*measure* (higher is better) averaged over 10 cross validations (with ranks in parentheses).

Data	*BR*	*SMBR*	*NLDD*	*ECC*	*RAKEL*	*HOMER*	*RF*-*PCT*	*MLKNN*	*CBM*
emotions	0.603 (7)	0.629 (5)	0.645 (3)	0.648 (2)	0.632 (4)	0.670 (1)	0.628 (6)	0.399 (9)	0.472 (8)
scene	0.625 (8)	0.643 (7)	0.736 (1)	0.715 (4)	0.692 (5)	0.716 (3)	0.595 (9)	0.683 (6)	0.731 (2)
yeast	0.609 (8)	0.616 (5)	0.644 (2)	0.647 (1)	0.625 (3)	0.562 (9)	0.622 (4)	0.614 (7)	0.615 (6)
medical	0.795 (6)	0.796 (5)	0.827 (2)	0.826 (3)	0.793 (7)	0.801 (4)	0.697 (8)	0.603 (9)	0.831 (1)
slashdot	0.503 (6)	0.516 (5)	0.562 (2)	0.561 (3)	0.502 (7)	0.528 (4)	0.220 (8)	0.073 (9)	0.567 (1)
enron	0.512 (8)	0.530 (4)	0.520 (7)	0.585 (1)	0.522 (5)	0.546 (3)	0.562 (2)	0.426 (9)	0.522 (6)
ohsumed	0.453 (7)	0.455 (6)	0.488 (4)	0.524 (1)	0.455 (5)	0.497 (2)	0.381 (8)	0.091 (9)	0.494 (3)
tmc2007	0.666 (4)	0.670 (3)	0.662 (6)	0.664 (5)	0.660 (7)	0.672 (2)	0.688 (1)	0.556 (9)	0.601 (8)
bibtex	0.397 (5)	0.393 (6)	0.411 (2)	0.406 (3)	0.402 (4)	0.323 (7)	0.190 (8)	0.160 (9)	0.437 (1)
av. ranks	6.6	5.1	3.2	2.6	5.1	3.9	6.0	8.4	4.0

According to the Friedman tests, the classifiers are not all equal (*p* < 0.05). The post-hoc analysis - adjusted for multiple testing - showed that *NLDD* performed significantly better than *BR* and *SMBR* on all metrics, significantly better than *RAKEL* and *MLKNN* on all but *Hammingloss*, significantly better than *HOMER* on *Hammingloss* and 0∕1*loss*, and significantly better than *ECC* and *RF*-*PCT* on 0∕1*loss*. No method performed statistically significantly better than *NLDD* on any evaluation metric.

*NLDD* achieved lowest (i.e., best) average ranks on 0∕1*loss* and *multi*-*label accuracy*, while *ECC* and *RF*-*PCT* achieved the lowest average ranks on the *F*-*measure* and *Hammingloss*, respectively. On both *F*-*measure* and *Hammingloss*, *NLDD* achieved the second lowest (i.e., best) average ranks. *CBM* achieved the second lowest average rank on 0∕1*loss* and *multi*-*label accuracy*. The performance of *CBM* on the 0∕1*loss* was very variable achieving the lowest rank on five out of nine data sets and the second worst on two data sets.

We next look at the performance of *NLDD* by whether or not the true labelsets were observed in the training data. A labelset has been observed if the exact labelset can be found in the training data and unobserved otherwise. Since *NLDD* makes a prediction by choosing a training labelset, a predicted labelset can only be partially correct on an unobserved labelset. Table 6 compares the evaluation results of *BR* and *NLDD* on two separate subsets of the test set of the *bibtex* data. The bibtex data were chosen because the data set contains by far the largest percentage of unobserved labelsets (33%) among the data sets investigated. The test data set was split into subsets *A* and *B*; if the labelset of a test instance was an observed labelset, the instance was assigned to *A*; otherwise the instance was assigned to *B*. For all of the four metrics, *NLDD* outperformed *BR* even though 33% of the labelsets in the test data were unobserved labelsets.

**Table 6:** Evaluation results on the bibtex data set by whether or not the labelset was observed (Subset *A*) or unobserved (Subset *B*) in the training data. Subset *A* contains 67% of the test instances and subset *B* contains 33%. For *Hammingloss* and 0∕1*loss*, lower is better. For *Multi*-*label accuracy* and *F*-*measure*, higher is better.

	Subset *A*		Subset *B*		Total (*A*∪*B*)	
	*BR*	*NLDD*	*BR*	*NLDD*	*BR*	*NLDD*
*Hammingloss*	0.0113	0.0091	0.0250	0.0224	0.0158	0.0134
0∕1*loss*	0.7804	0.7163	0.9958	1.0000	0.8504	0.8084
*Multi*-*labelaccuracy*	0.3807	0.4273	0.2118	0.1870	0.3259	0.3492
*F*-*measure*	0.4402	0.4785	0.3065	0.3058	0.3966	0.4130

We next look at the three regression parameters the proposed method (*NLDD*) estimated (Eq. (2)) for each data set in more detail. Table 7 displays the *MLE* of the parameters of the binomial model in each data set. In all data sets, the estimates of *β*_1_ and *β*_2_ were all positive. The positive slopes imply that the expected loss (or, equivalently the probability of misclassification for each label) decreases as ${D}_{\vec{x}}$ or ${D}_{\vec{y}}$ decreases.

**Table 7:** The maximum likelihood estimates of the parameters of equation (2) averaged over 10 cross validations.

Data	${\hat {\beta }}_{0}$	${\hat {\beta }}_{1}$	${\hat {\beta }}_{2}$
emotions	−2.6353	0.0321	1.0912
scene	−3.5023	0.0134	1.8269
yeast	−3.9053	0.1409	0.8546
medical	−5.5296	0.1089	1.6933
slashdot	−4.2503	0.1204	1.3925
enron	−3.8827	0.0316	0.7755
bibtex	−4.8436	0.0093	0.7264
ohsumed	−3.1341	0.0022	0.9855
tmc2007	−3.6862	0.0370	1.1056

From the values of ${\hat {\beta }}_{0}$ we may infer how low the expected loss is when either ${D}_{\vec{x}}$ or ${D}_{\vec{y}}$ is 0. For example, ${\hat {\beta }}_{0}=-3.5023$ in the *scene* data set. If ${D}_{\vec{x}}=0$ and ${D}_{\vec{y}}=0$, $\hat {p}=0.0292$ because $\log \frac{\hat {p}}{1-\hat {p}} =-3.5023$. Hence $\hat {E}(loss)=L\hat {p}=6\cdot 0.0292=0.1752$. This is the expected number of mismatched labels for choosing a training labelset whose distances to the new instance are zero in both feature and label spaces. The results suggest the expected loss would be very small when classifying a new instance that had a duplicate in the training data (${D}_{\vec{x}}=0$) and whose labels are predicted with probability 1 and the predicted labelset was observed in the training data (${D}_{\vec{y}}=0$).

## Scaling up *NLDD*

As seen in ‘Nearest labelset using double distances (*NLDD*)’, the time complexity of *NLDD* is dependent on the size of the training data (*N*). In particular, the term *O*(*N*^2^(*d* + *L*)) makes the complexity of *NLDD* quadratic in *N*. For larger data sets the running time could be reduced by running the algorithm on a fraction of the *N* instances, but performance may be affected. This is investigated next.

**Figure 3:**
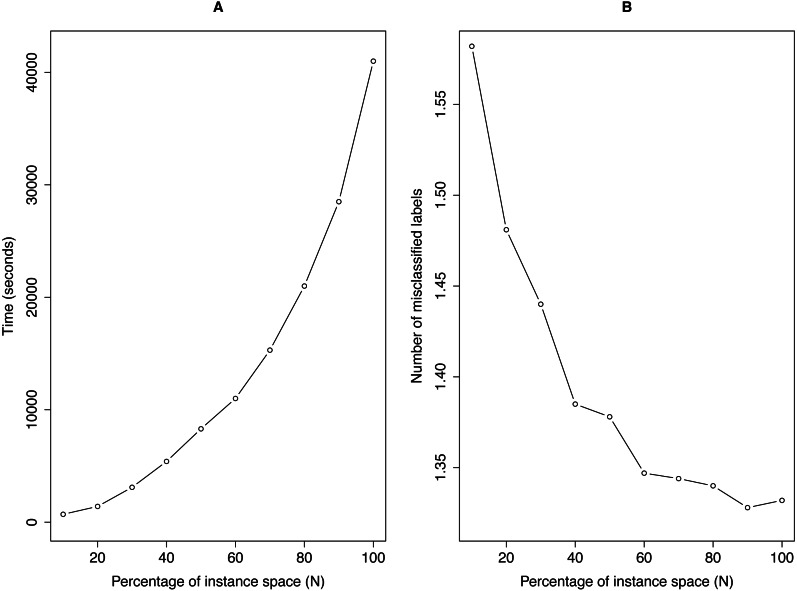
Running time (A) and the average number of mismatched labels (B) as a function of the percentage of the instance space for *NLDD*.

Figure 3 illustrates the running time and the corresponding performance of *NLDD* as a function of the percentage of *N*. For the result, we used the *tmc*2007 data with 75/25 train/test splits. After splitting, we randomly chose 10%–100% of the training data and ran *NLDD* with the reduced data. As before, we used *SVM* with a linear kernel as the base classifier.

The result shows that *NLDD* can obtain similar predictive performances for considerably less time. The running time increased quadratically as a function of *N* while the improvement of the performance of *NLDD* appeared to converge. Using 60% of the training data, *NLDD* achieved almost the same performance in the number of mismatched labels as using the full training data. Similar results were obtained on other large data sets.

## Discussion

For the sample data sets selected, *NLDD* achieved the lowest (i.e., best) average ranks on 0∕1*loss* and *multi*-*label accuracy*, and the second lowest average ranks on *Hammingloss* and *F*-*measure* compared with other state-of-art methods.

What may explain the success of *NLDD*? *NLDD* minimizes a function of two distances. *NLDD* performs substantially better than separate approaches that rely on only one of the distances: *k*-nearest neighbors (*k* = 1) using ${D}_{\vec{x}}$ only or *SMBR* using ${D}_{\vec{y}}$ only (Supplemental Information). *NLDD* integrates the two distances using an additive model Eq. (2). The specific integration does not appear crucial: we have experimented with a multiplicative model, $\log \left( \frac{\theta }{1-\theta } \right) ={\beta }_{0}+{D}_{\vec{x}}^{{\beta }_{1}}{D}_{\vec{y}}^{{\beta }_{2}}$, that performed similarly (results not shown). Therefore the success seems due to the combination of two quite different distances. The distances may be complementary in that ${D}_{\vec{x}}$ corresponds to a highly local classifier (*kNN* with *k*=1) and ${D}_{\vec{y}}$ draws on a global classifier. Computing the distance ${D}_{\vec{y}}$ requires estimating the probability of each label using a base classifier. The classifier used here, *SVM*, is a general global classifier. Some evidence for the conjecture that a global base classifier is important are experiments using nearest neighbors (*kNN*) instead of *SVM* as a base classifier: a more global choice (*k* = 30) yielded much improved results over a more local choice (*k* = 3) (Supplemental Information).

Like *BR*,   *NLDD* uses outputs of independent binary classifiers. Using the distances in the feature and label spaces in binomial regression, *NLDD* can make more accurate predictions than *BR*. *NLDD* was also significantly superior to *SMBR*, which is similar to *NLDD* in the sense that it makes predictions by choosing training labelsets using binary classifiers. One of the reasons why *NLDD* performs better than *BR* and *SMBR* is that it contains extra parameters. *SMBR* is based on the label space only, while *NLDD* uses the distances in the feature space as well.

Like *LP*, the proposed method predicts only labelsets observed in the training data. In restricting the labelsets for prediction, higher order correlations among the labels are implicitly accounted for. At the same time, this restriction is *NLDD*’s main limitation. If a new instance has a true labelset unobserved in the training data, there will be at least one incorrectly predicted label. Even so, *NLDD* scored best on two metrics and second best on two other metrics. How frequently an unobserved labelset occurs depends on the data set. For most data sets, less than 5% of the test data contained labelsets not observed in the training data. In other words, most of the labelsets of the test instances could be found in the training data. However, for the bibtex data set about 33% of the test data contained unobserved labelsets. As seen in Table 6, when the true labelsets of the test instances were not observed in the training data (subset *B*), *BR* performed slightly better than *NLDD* in terms of 0∕1*loss*, *multi*-*label accuracy* and *F*-*measure*. On the other hand, when the true labelsets of the test instances were observed in the training data (subset *A*), *NLDD* outperformed *BR* on all of the metrics. Combined, *NLDD* achieved higher performances than *BR* on the entire test data. However, *NLDD* might not fare as well when the percentage of unobserved labelsets is substantially greater.

The use of binomial regression (see equation Eq. (2)) implies that the misclassification probability *θ* is constant for each label. Although the true misclassification probabilities may differ for labels, the experimental results showed that *NLDD* performs well under this assumption. Instead of using binomial regression and estimating a single constant *θ*, one might have used *L* logistic regressions to estimate individual *θ*_*i*_ (*i* = 1, …, *L*) for each label. Rather than choosing the labelset that minimizes a single *θ*, one could have then chosen the labelset that minimizes a function of the *θ*_*i*_. However, choosing such a function is not straightforward. Also, this requires estimating 3*L* parameters instead of 3.

*NLDD* uses binomial regression to estimate the parameters. This setup assumes that the instances in *S* are independent. While it turned out that this assumption worked well in practice, dependencies may arise between the two pairs of a given *S*_*i*_. If required this dependency could be modeled using, for example, generalized estimating equations (*GEE*) (Liang & Zeger, 1986). We examined *GEE* using an exchangeable correlation structure. The estimates were almost the same and the prediction results were unchanged. The analogous results are not shown.

*NLDD* has higher time complexity than *BR*. The relative differences of running time between *NLDD* and *BR* depended on the size of the training data (*N*). The number of labels and features had less impact on the differences, as the complexity of *NLDD* is linear in them.

For prediction, the minimization in Eq. (3) only requires the estimates of the coefficients *β*_1_ and *β*_2_ which determine the tradeoff between ${D}_{\vec{x}}$ and ${D}_{\vec{y}}$. The estimate of *β*_0_ is not needed. However, estimating *β*_0_ allows us to also estimate the probability of a misclassification of a label for an instance, $\hat {\theta }$. Such an assessment of uncertainty of the prediction can be useful. For example, one might only want to classify instances where the probability of misclassification is below a certain threshold value.

*NLDD* uses a linear model for binomial regression specified in Eq. (2). To investigate how the performance of *NLDD* changes in nonlinear models, we also considered a model: $\log \left( \frac{\theta }{1-\theta } \right) ={\beta }_{0}+{D}_{\vec{x}}^{{\beta }_{1}}\cdot {D}_{\vec{y}}^{{\beta }_{2}}$ in which the distances are combined in a multiplicative way. The difference of prediction results obtained by the linear and multiplicative models was small.

While we used the Euclidean distance for *NLDD*, other distance metrics such as the Manhattan distance may also be employed. We ran *NLDD* based on the Manhattan distance in the label space and the results were almost the same: over 99% of the prediction were identical and the differences of the performance in all metrics were less than 1%(the Euclidean distance gave slightly better performance for most data). This shows that the difference in prediction performance between the Manhattan and the Euclidean metrics was tiny in practice.

While *SVM* was employed as the base classifier, other algorithms could be chosen provided the classifier can estimate posterior probabilities rather than just scores. Better predictions of binary classifiers will make distances in the label space more useful and hence lead to a better performance.

Lastly, we observed that the distributions of labels are, in general, unbalanced for many multi-label datasets. Since the performance of traditional classification algorithms can be limited on unbalanced data, addressing this problem could improve the reliability of the probabilistic classifiers, and result in an improved performance of *NLDD*. To mitigate the unbalanced distributions of labels, we applied Synthetic Minority Over-sampling Technique (*SMOTE*) (Chawla et al., 2002) that evens out the class distribution by generating synthetic examples of the minority class. Probabilistic classifiers were then trained on the expanded training data and used in the process of *NLDD*. For 7 out of the 9 data sets, the 0∕1*loss*, *multi*-*label accuracy* and *F*-*measure* were improved by a modest amount.

## Conclusion

In this article, we have presented *NLDD* based on probabilistic binary classifiers. The proposed method chooses a training labelset with the minimum expected loss, where the expected loss is a function of two variables: the distances in feature and label spaces. The parameters are estimated by maximum likelihood. The experimental study with nine different multi-label data sets showed that *NLDD* outperformed other state-of-the-art methods on average in terms of 0∕1*loss* and *multi*-*label accuracy*.

##  Supplemental Information

10.7717/peerj-cs.242/supp-1 Supplemental Information 1 Effect of global and local base classifiers on the performance of NLDD The figure shows the percentage of improvement of NLDD over BR in terms of Hamming loss and 0/1 loss in all data sets. For the base classifier, two variations of *k* nearest neighbor (kNN) are used: a more global choice (*k* = 30) and a more local choice (*k* = 3).

10.7717/peerj-cs.242/supp-2 Supplemental Information 2 Effect of global and local base classifiers on the performance of NLDD The figure shows the percentage of improvement of NLDD over BR in terms of multi-label accuracy and F-measure in all data sets. For the base classifier, two variations of *k* nearest neighbor (kNN) are used: a more global choice (*k* = 30) and a more local choice (*k* = 3).

10.7717/peerj-cs.242/supp-3 Table S1 Comparison of NLDD with NN and SBMR in terms of Hamming loss Table S1 compares NLDD with two other approaches that rely on only one of the distances: nearest neighbor (NN) using Dx only and SMBR using Dy only.

10.7717/peerj-cs.242/supp-4 Table S2 Comparison of NLDD with NN and SBMR in terms of 0/1 loss Table S2 compares NLDD with two other approaches that rely on only one of the distances: nearest neighbor (NN) using Dx only and SMBR using Dy only.

10.7717/peerj-cs.242/supp-5 Table S3 Comparison of NLDD with NN and SBMR in terms of multi-label accuracy Table S3 compares NLDD with two other approaches that rely on only one of the distances: nearest neighbor (NN) using Dx only and SMBR using Dy only.

10.7717/peerj-cs.242/supp-6 Table S4 Comparison of NLDD with NN and SBMR in terms of F-measure Table S4 compares NLDD with two other approaches that rely on only one of the distances: nearest neighbor (NN) using Dx only and SMBR using Dy only.

## Additional Information and Declarations

### Data Availability

The following information was supplied regarding data availability: Raw data is available at Github: https://github.com/hgweon/HG-multilabel.

## References

[ref-1] Blockeel H, Raedt L, Ramon J (1998). Top-down induction of clustering trees.

[ref-2] Boutell MR, Luo J, Shen X, Brown CM (2004). Learning multi-label scene classification. Pattern Recognition.

[ref-3] Chawla NV, Bowyer KW, Hall LO, Kegelmeyer WP (2002). SMOTE: synthetic minority over-sampling technique. Journal of Artificial Intelligence Research.

[ref-4] Demšar J (2006). Statistical comparisons of classifiers over multiple data sets. Journal of Machine Learning Research.

[ref-5] Elisseeff A, Weston J (2001). A kernel method for multi-labelled classification.

[ref-6] Godbole S, Sarawagi S, Dai H, Srikant R, Zhang C (2004). Discriminative methods for multi-labeled classification. Advances in knowledge discovery and data mining.

[ref-7] Gonçalves T, Quaresma P (2003). A preliminary approach to the multilabel classification problem of portuguese juridical documents.

[ref-8] Hersh W, Buckley C, Leone TJ, Hickam D (1994). OHSUMED: an interactive retrieval evaluation and new large test collection for research.

[ref-9] Hochberg Y (1988). A sharper Bonferroni procedure for multiple tests of significance. Biometrika.

[ref-10] Ji S, Tang L, Yu S, Ye J (2008). Extracting shared subspace for multi-label classification.

[ref-11] Katakis I, Tsoumakas G, Vlahavas I (2008). Multilabel text classification for automated tag suggestion.

[ref-12] Klimt B, Yang Y (2004). The enron corpus: a new dataset for email classification research.

[ref-13] Kocev D, Vens C, Struyf J, Džeroski S (2007). Ensembles of multi-objective decision trees.

[ref-14] Li C, Wang B, Pavlu V, Aslam JA (2016). Conditional Bernoulli mixtures for multi-label classification.

[ref-15] Li T, Ogihara M (2003). Detecting emotion in music.

[ref-16] Liang K-Y, Zeger SL (1986). Longitudinal data analysis using generalized linear models. Biometrika.

[ref-17] Liu F, Zhang X, Ye Y, Zhao Y, Li Y (2015). MLRF: multi-label classification through random forest with label-set partition.

[ref-18] Madjarov G, Kocev D, Gjorgjevikj D, Džeroski S (2012). An extensive experimental comparison of methods for multi-label learning. Pattern Recognition.

[ref-19] Meyer D, Dimitriadou E, Hornik K, Weingessel A, Leisch F (2014). http://CRAN.R-project.org/package=e1071.

[ref-20] Platt J, Smola A, Bartlett P, Schoelkopf B, Schuurmans D (2000). Probabilistic outputs for support vector machines and comparisons to regularized likelihood methods. Advances in large margin classifiers.

[ref-21] R Core Team (2014). http://www.R-project.org/.

[ref-22] Read J, Pfahringer B, Holmes G (2008). Multi-label classification using ensembles of pruned sets.

[ref-23] Read J, Pfahringer B, Holmes G, Frank E (2011). Classifier chains for multi-label classification. Machine Learning.

[ref-24] Schapire RE, Singer Y (1999). Improved boosting algorithms using confidence-rated predictions. Machine Learning.

[ref-25] Schapire RE, Singer Y (2000). BoosTexter: a boosting-based system for text categorization. Machine Learning.

[ref-26] Srivastava A, Zane-Ulman B (2005). Discovering recurring anomalies in text reports regarding complex space systems.

[ref-27] Tai F, Lin H-T (2012). Multilabel classification with principal label space transformation. Neural Computation.

[ref-28] Trohidis K, Tsoumakas G, Kalliris G, Vlahavas I (2008). Multilabel classification of music into emotions.

[ref-29] Tsoumakas G, Katakis I (2007). Multi-label classification: an overview. International Journal of Data Warehousing and Mining.

[ref-30] Tsoumakas G, Katakis I, Vlahavas I (2008). Effective and efficient multilabel classification in domains with large number of labels.

[ref-31] Tsoumakas G, Katakis I, Vlahavas I, Maimon O, Rokach L (2010). Mining Multi-label Data. Data mining and knowledge discovery handbook.

[ref-32] Tsoumakas G, Katakis I, Vlahavas I (2011). Random k-Labelsets for multilabel classification. IEEE Transactions on Knowledge and Data Engineering.

[ref-33] Tsoumakas G, Vlahavas I (2007). Random k-labelsets: an ensemble method for multilabel classification.

[ref-34] Vapnik VN (2000). The nature of statistical learning theory.

[ref-35] Wilcoxon F (1945). Individual comparisons by ranking methods. Biometrics Bulletin.

[ref-36] Ypma TJ (1995). Historical development of the Newton-Raphson method. SIAM Review.

[ref-37] Zhang M-L, Zhou Z-H (2005). A k-nearest neighbor based algorithm for multi-label classification.

[ref-38] Zhang M-L, Zhou Z-H (2007). ML-KNN: a lazy learning approach to multi-label learning. Pattern Recognition.

[ref-39] Zhang ML, Zhou ZH (2014). A review on multi-label learning algorithms. IEEE Transactions on Knowledge and Data Engineering.

