# Supplementary figures and images for: Nearest labelset using double distances for multi-label classification
Source: PeerJ Comput Sci. 2019 Dec 9;5:e242. doi: 10.7717/peerj-cs.242 (PMC7924696; doi:10.7717/peerj-cs.242)

### Hamming loss

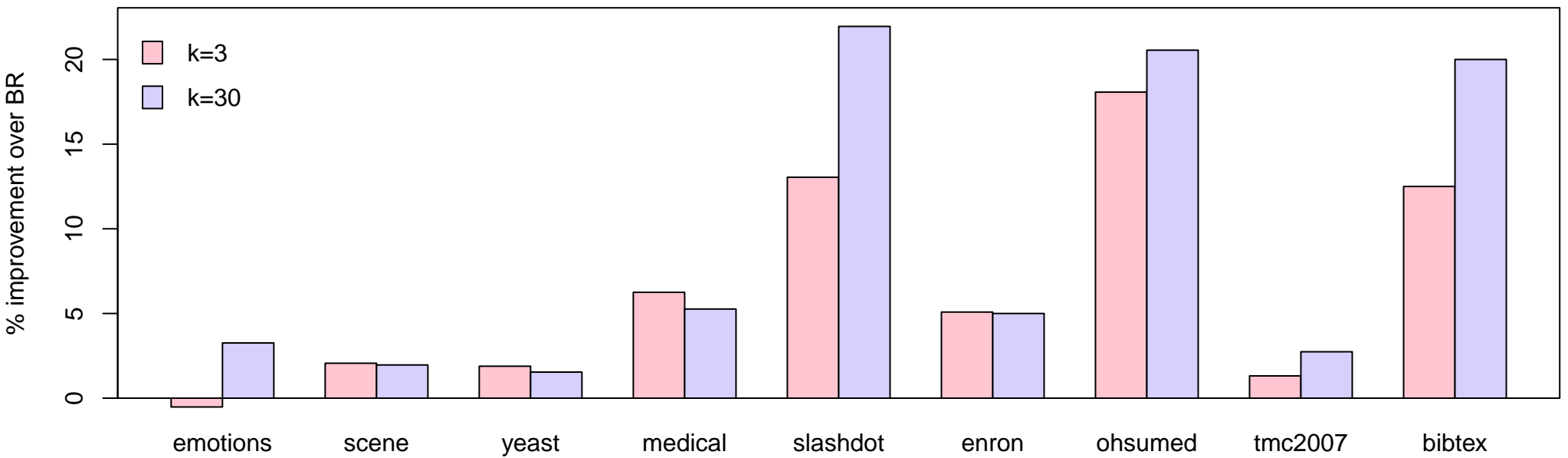

### 0/1 loss

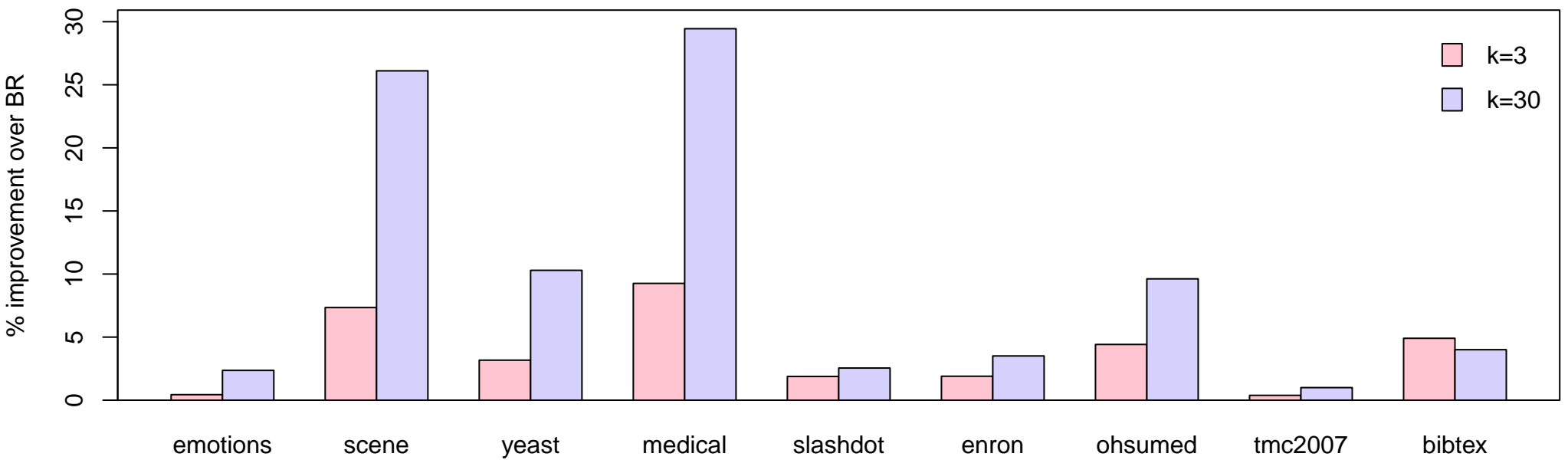

Supplement: Supplemental Information 1 — The figure shows the percentage of improvement of NLDD over BR in terms of Hamming loss and 0/1 loss in all data sets. For the base classifier, two variations of k nearest neighbor (kNN) are used: a more global choice (k = 30) and a more local choice (k = 3). [file peerj-cs-05-242-s001.pdf]

**Multi-label accuracy**

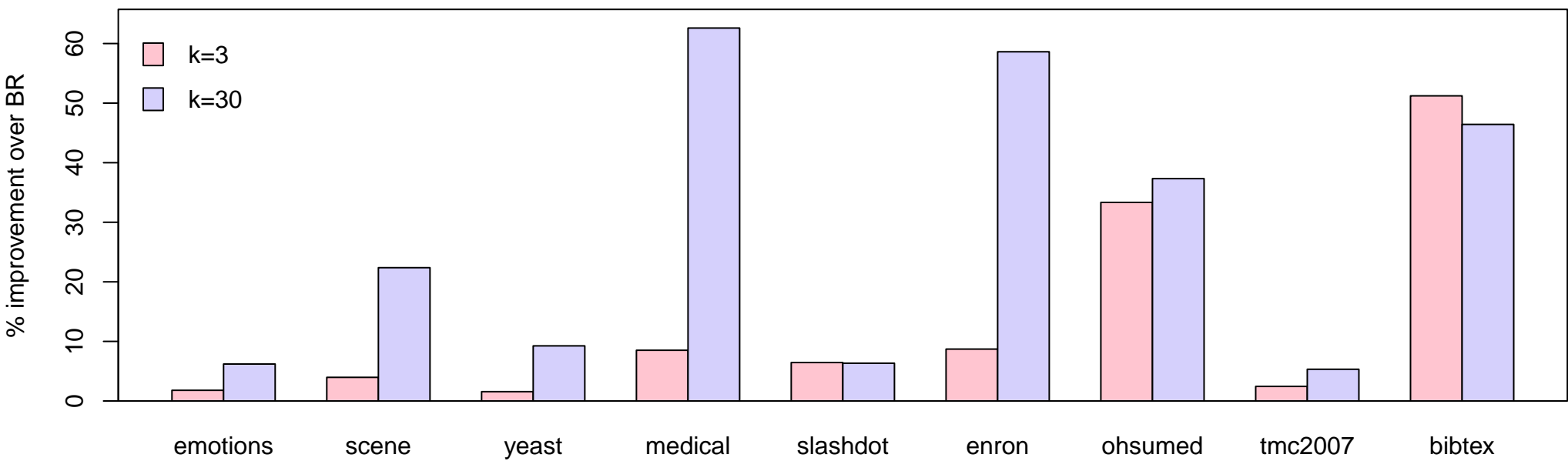

**F-measure**

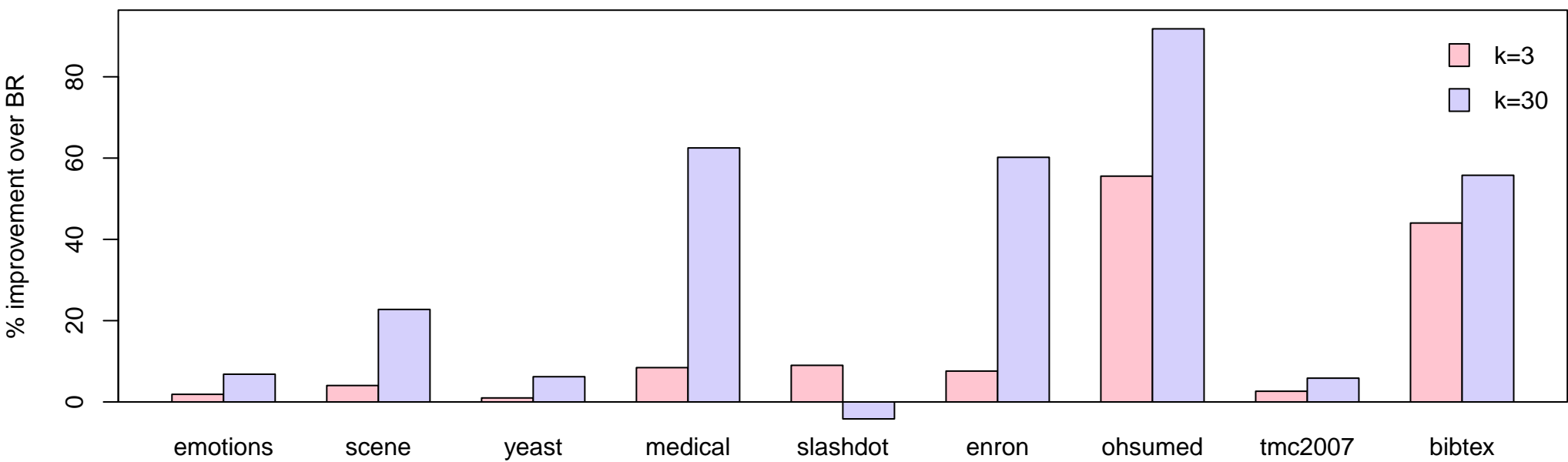

Supplement: Supplemental Information 2 — The figure shows the percentage of improvement of NLDD over BR in terms of multi-label accuracy and F-measure in all data sets. For the base classifier, two variations of k nearest neighbor (kNN) are used: a more global choice (k = 30) and a more local choice (k = 3). [file peerj-cs-05-242-s002.pdf]
